# On the Nature, Causes, Prevention, and Treatment of Acute Hydrocephalus

**Published:** 1845-10

**Authors:** 


### ON THE NATURE, CAUSES, PREVENTION AND TREATMENT OF ACUTE HYDROCEPHALUS, OR WATER-BRAIN FEVER. By *Thomas Smith*, M.D. 8vo. pp. 168. London, Longmans, 1845.

HYDROCEPHALUS is so frequent and so destructive a disease that a new publication concerning it is sure to challenge due attention. The present Essay, however, is unfortunately addressed to both the general and the professional reader, the inevitable consequence being, that it treats of topics which the former can neither comprehend, nor would benefit by comprehending, while it contains but little allusion to various points respecting which the latter is anxious for additional information. We hold that attempts at the instruction of the public in the "nature, causes, prevention, and treatment" of important diseases are not only useless, but fraught with danger. They beget an injudicious meddling and self-sufficiency in some minds, and needless and nervous apprehensions in others, by which the unfortunate little patient is often driven into the very evils sought to be averted. Before saying more upon the subject, it will be better to place the view Dr. Smith takes of it before our readers.

"If a popular treatise on any malady or class of maladies be desirable, it will surely be so on one which, when once established in its most decided form, is generally admitted to be unmanageable and without hope. It is because hydrocephalus is a desperate, and but too frequently, an incurable malady, that, so far as I am able to form an opinion, it is of the utmost importance that the medical attendant, the parent, and the nurse, should be on the alert to watch the threatening signs of its insidious approach, and by a seasonable interposition of diet, regimen, and remedy, to oppose the mischief at its onset. It may be stated, without hazarding any doubtful responsibility, that this fatal and rapidly growing disease ought not to be ranked with those whose progressive advance can neither be seen nor prevented. It very seldom sets in but as the result of a previous and gradual change in the condition of the child's system, tolerably manifest to the observation of an attentive parent or nurse; yet, how frequently little or nothing is done to arrest its further progress, until the active form presents itself; and then the family medical attendant has barely to announce the *name* of the disease, and every mother at once recognizes the knell of death in the sound.

"To the most severe and uncompromising critic, who may ask to what good end this, my effort, may lead, my answer is, I have found medical and popular literature equally deficient in suitable admonitions to parents and nurses. I have found statistics telling us that one-eighth of infantile deaths in crowded towns have occurred from water-brain-fever. I have found my own profession, with regard to the disease in its least equivocal form, confessing the impotence of medicine, and physicians pursuing, as the result of habit, the same uniform, authorized, but unsuccessful treatment of the disease, as on a mere routine without hope, without encouragement, without comfort, and without consolation; meeting in consultation in such cases merely to reflect reciprocal humiliation, and to indulge in reciprocal confessions of incompetency. I have found them persisting in a plan of treatment in which generation has servilely imitated generation, and under which an occasional recovery has been more a matter of wonder, and occurring more from the intervention of some unexpected constitutional effort in the patient, than from any well directed aim of science. * * *

"In such a position, after duly weighing all authorities, and having had many opportunities of comparing my sentiments with those of my contemporaries, if I have determined to try to lay down a *beacon* or two, by means of which even a few lives may be saved, and much misery prevented, it ought rather to excite surprise that I have not long ago been anticipated in the performance of what may justly be called a duty of humanity, long neglected. And although others after me may perform the same duty more ably, and perhaps more successfully, I may at least claim to myself the credit of first pointing the way." P. v.

After the perusal of the above rather grandiloquent introductory passage, we turned to the chapters on the Means of Prevention, naturally expecting to find some novel suggestions or improved prophylaxis; but great was our disappointment, since we found nothing there but has been more fully and better told by Dewees, Eberle, Evanson and Maunsell, Barlow, Combe, and numerous other writers, who have of late years treated upon the Physical Management of Children. What the author means by saying he is the first to lay down the beacons derivable from these instructions we know not. He may be the first who has engrafted them upon a professional treatise on hydrocephalus intended for popular perusal, and thereby seems to us to be running far more risk of planting rocks than of raising beacons. Professional men are well engaged in endeavouring to impart to the public approved rules for the preservation of health—the principles of hygiene—insisting that recourse should be had to their assistance when any aberration from the healthy condition occurs. The author may tell us that his work is addressed to this end; but if so, why couple with his hygienic precepts, which are just as applicable to the prevention of any other diseased condition as this, an account of the symptoms, diagnosis, treatment, &c. of hydrocephalus. When we consider the difficulty that frequently exists in the detection of this disease, and the errors able and practical men not unfrequently fall into respecting it, the scheme of enabling nurses, parents, and the like, to compass this by furnishing them with an accurate account of its well-marked form, seems to us, to say the least, a very foolish one. If Dr. Smith believes these personages will confine their attention to the portion of the work suited to their capacity, he can know little of the habits of the class whose instruction he is labouring for; for however inapplicable the maxim "that a little knowledge is a dangerous thing," may be in general, we have had too often to deplore their meddlings and thwartings to doubt its infallibility as regards the management and treatment of disease. The arguments for endeavouring to popularize the recognition and management of this disease are just the same fallacious ones the notorious Buchan—whose work has consigned thousands to their graves—employed long ago.

Although the term Hydrocephalus is a defective one, as only indicating one of the effects of the diseased condition, it is perhaps not very easy to substitute a better; and indeed, when employed in the sense which Heberden first attached to the term dropsy, suffices well enough—"*Hydrops non tam ipse morbus est quam alicujus morbi signum.*" Dr. Hennis Green, and the writers of the French school who have succeeded him, term the disease *Tubercular Meningitis,* on account of the very great frequency with which tubercles, or lardaceous deposits are found in the inflamed pia mater. Of 60 cases observed at the Children's Hospital at Paris, Dr. Green found these appearances in 56. MM. Rilliet and Barthez found the same 29 times out of 33 cases, and M. Bouchut in six out of nine cases. This complication, however, does not by any means so frequently occur in this country as in France; and the exceptions even allowed by the above-named authors should suffice to prevent this term being accepted. However this may be, the intimate connection of the disease with the *strumous diathesis* has been long observed. The observations of English, German, and French writers, but especially the latter, all tend to show the rarity of the occurrence of hydrocephalus without tuberculization of some one or more organs of the economy. Still there can be no doubt that cases of this disease do occasionally occur in subjects unaffected with tubercle, it being then a simple inflammatory disease, which may come on primarily in an otherwise healthy subject, or secondarily as a consequence of scarlatina, &c. In the vast majority of cases it is the strumous and far less manageable form of inflammatory action which prevails. Dr. Smith considers the disease as analogous to the *nervous fever* of adults, and, but for the inconvenience of changing names, would term it *infantile nervous fever*, or, as he expresses it on the title-page of his work, "*water-brain fever.*"

*Symptoms and Stages.*—"In 1768," MM. Rilliet and Barthez observe, "appeared Robert Whytt's remarkable dissertation, and as regards the symptomatology it is even now the best work that has ever yet appeared. We cannot too much admire the talent for description, and the eminent spirit of observation which shine through every page of this interesting monograph; nor praise too much the ideas, full of justice, by which the author seeks to explain the development of the different symptoms." Whytt divided this disease into three stages, the varying state of the pulse forming a principal indication; this being rapid in the first or precursory, and in the last or convulsive stage, and slow in the second stage, when stupor first commences. To this there are exceptions; and, although his arrangement has been generally followed, it would be preferable, M.

Bouchut observes, to take into account the *ensemble* of the symptoms rather than any one. This writer terms the three stages Germination (which distinguishes the disease from simple meningitis of infants), Invasion and Convulsion. MM. Rilliet and Barthez seem to doubt the expediency of this division into stages, observing that the disease in all their cases manifested continuity; one or more symptoms may have remitted, but this has never been the case with all. Dr. Smith adopts the division of Whytt into three stages, that of excitement, of effusion, and of convulsion, attributing a medium duration to the whole of them of about 21 days; or, dating from when the child first manifested feverishness, change of temper, &c. of from four to six weeks. Of 117 cases, Dr. Green observed death to occur within 7 days in 31; 14 days in 49; 20 days in 31; more than 20 days in 6. MM. Rilliet and Barthez have never seen death cccur before the 7th day, and usually between the 11th and 20th. The premonitory symptoms are accurately but cursorily described by Dr. Smith, who observes that "any intelligent parent or nurse may remark the gradual change taking place in such infants." They may observe this change, but will often, as experienced observers even have done, mistake other affections for it; and when they do recognize it, we believe the author is too sanguine in assuring them that farther mischief may be arrested; for we fear there is too much truth in Dr. Hennis Green's opinion, that these symptoms are indications of the presence of chronic meningeal inflammation—too often irremediable.

*The Pulse.*—MM. Rilliet and Barthez state there is nothing to be added to Whytt's observations, which only require slight modification. They observe:

"The acceleration is of short duration, and in a few days the pulse does not fail to offer the normal number of beats, or even fall below these, this diminution continuing for a variable period, but always in relation to the duration of the disease. So that, if the pulse only beats 60, 80, or 90, we may be certain that the disease will be prolonged for some days; but as soon as a new acceleration occurs we must regard a fatal termination as approaching, within the limits of from two to three days, or in some exceptional cases between five and six. When the pulse recommences quickening it generally beats from 112 to 120, and if this occurs on the very day of death from 140 to 160. In the cases where the increase occurs at a considerable period prior to the fatal termination, there is a continued progression until that moment arrives. The rapidity of the pulse is such then as hardly to be measured; we have counted it from 192 to 200 the evening before or day of death. * * * We have so much insisted on the character of the pulse, because, from among a considerable number of diseases of every kind, simple or complicated, acute or chronic, we have hardly ever found the pulse at once slow and irregular, except in the tuberculo-inflammatory affections of the brain and its dependences."

Whytt states that the pulse is from 130 to 160 at the commencement of the affection: but the observations of Dr. Hennis Green are quite at variance with this statement. According to this talented author, the pulse is always *slow* at the commencement of the disease, *i. e.* between the period of the occurrence of headache and vomiting, and that of somnolency. Of 19 cases observed, with reference to this point, in two only did it exceed 100. It averaged 80, and in some was as low as 54.

Dr. Smith observes:—

"In Hydrocephalus the pulse during the first stage is much accelerated, full, but more easily compressible than in encephalitis, with a perceptible variation in the rhythm of the artery, and in the regularity of the strokes. The pulse sometimes beats as quick during one-third of a given time as it had previously done during the former two-thirds. Thus, in a child whose pulsations were 140 a minute, I have witnessed half, or 70 strokes, performed in 40 seconds, and the remainder in the other 20 seconds. There is also a distinct intermittency in the 7, 17, or 20 pulsation, and it often changes its character; one or two strokes, in quick succession, may be felt soft, weak, and fluttering. In the second stage, the pulse sinks and becomes slow, laboured, intermittent, and irregular, and is easily quickened by motion, or mental disturbance, to double its amount of pulsations." P. 31.

As to the symptoms referrible to the abdominal organs; the *appetite* is seldom entirely lost. Every author since the time of Whytt has acknowledged the importance of *vomiting* as a symptom. Rilliet and Barthez observe that it generally occurs on the first day, and very rarely later than the second or third, usually continuing only for two or three days, although at other times for a much longer period, being very seldom reproduced after disappearing. M. Piet thus states the value attached to this sign: "If, in a child that has been vaccinated or had the small-pox, who digests well, and is suffering neither from bronchitis or pertussis, vomiting, whether simple or bilious, occurs, accompanied or preceded by a more or less continued headache, there is every reason to fear an approaching meningitis, especially if the child is tuberculous." Dr. Smith observes that it is usually spontaneous and unattended with nausea, while, after its occurrence, food is often taken with avidity. The value of vomiting as a symptom becomes of much more importance when conjoined with *constipation*. This, although a frequent, is not a constant symptom, but is of very difficult removal when it does occur. Diarrhœa, however, usually supervenes before the termination of the disease. Another symptom usually occurring about the sixth day, but sometimes later, is a *retraction of the walls of the abdomen*. MM. Rilliet and Barthez observe—"The belly becomes depressed at its centre and takes the form of a boat; the contraction being sometimes carried so far as to allow the beating of the aorta to be felt. It is almost a constant symptom, and does not depend on the constipation, as it often comes on when diarrhœa replaces this. It is almost exclusively in cerebral affections we have observed this symptom." Of the *urine* in hydrocephalus Dr. Smith observes:—

"The urine for the most part is of a deep amber hue, of high specific gravity, sometimes milky, and deposits a whitish slimy sediment, smells offensively shortly after being passed, and in its passage along the urethra occasions pain. The character of the urine in Hydrocephalus is essentially different both in its composition and appearance from that evacuated in phrenitis or inflammation of the brain. In the latter disease, it is generally of a dark brown or porter colour, and contains more urea, and less of the lithates than the former. The deposit also is mostly of a reddish or reddish-brown hue." P. 9.

We need not enter into any detail of the cephalic symptoms, extracting however, the following passage upon that of *coma*, from the treatise of MM. Rilliet and Barthez:

"Is there any relation between the anatomical lesions and the coma? In the three cases in which coma and somnolence were entirely absent, we twice found a considerable inflammation of the pia mater at the base, and in the other case innumerable granulations, and a superficial ramollissement of the grey substance. The ventricular effusion was slight, about an ounce or two in two of the children and was quite absent in the other. Is it to the slight quantity or absence of this fluid we are to attribute the absence of the coma? In looking over other of our observations, we find the quantity of effusion not to be more considerable, and yet the coma complete. In all the other cases where it has been profound, we have found a considerable ventricular effusion at the autopsy."

Dr. Smith makes a remark or two upon the *cough* so commonly met with in Hydrocephalus.

"A slight false cough, which partly resembles a suppressed effort to vomit, and not unlike the morning liver cough of spirit drinkers, is not an infrequent attendant throughout the three stages. Alibert notices this symptom in conjunction with embarrassed breathing, as a sign of ventricular effusion. In vain will the practitioner try to relieve this cough with anodynes and demulcents; and it may be, that the chest shall be charged as the seat of the mischief, and the treatment, of a bronchitis or infantile pneumonia, shall be insisted on, whereas, it is but a sign of a certain condition of the brain and nervous system. * * * * * The cause of the cough above alluded to, appears to me to arise from irritation of the cerebral extremities of the pneumogastric nerve. Hence the various anomalous symptoms which occur in the stomach, diaphragm, liver, &c. indicated by vomiting, sighing, sobbing, and epigastric tenderness." P. 13.

*Diagnosis.*—We entirely agree with Dr. Smith in his opinion, that those practitioners who boast so much of their success in treating this affection, have mistaken phrenitis or some other form of disease for it. Many of the observations of this chapter would have found a more proper place in that treating of the symptoms of the disease. We will extract a few of those which are more properly speaking diagnostic. The *headache* of hydrocephalus is of a lancinating, paroxysmal character, and accompanied by involuntary ejaculations. "If desired to shake the head, the child usually essays the attempt, and suddenly stops, uttering the most piercing cry, and holds the head fast between the hands. The head feels heavy and full anteriorly, and there is a strong tendency to rest its frontal surface against the pillow." *Vertigo* is of more gradual approach and longer duration than in phrenitis, and is especially felt on assuming the erect posture. The *stupor* is usually more intense and longer continued than in other cerebral affections. Rheumatic and colicky pains are found oftener in the *abdomen,* together with tenderness along the cervical and dorsal vertebræ. They increase as the disease advances, effecting especially the hypogastric and epigastric regions. The *respiration* and *pulse* do not observe their usual consentaneous action. The pulse may beat from 140 to 160 per minute, while the respiratory movements do not exceed 40 or 50. "The respiratory effort is, for the most part, performed in a quick, hurried, convulsive manner, and there is a marked increase in the duration of the expiratory act and the period of repose; deep and prolonged sighing often intervenes, and considerably diminishes the amount of the respiratory movements." Dr. Smith thinks that late writers have done wrong in slighting the diagnostic value of the appearance of the

*fæces*, taken in connexion with the obstinate constipation which also prevails. "Compare the evacuations of a patient in phrenitis, small-pox, or remittent fever; do we find them possessing the same tenaceous, gluey, glossy, properties, and cadaverous smell, which characterize the stools in water-brain fever? The fæces in all these affections may, it is true, be offensive, but are they, at the same time, gelatinous or glairy?" The *eye* is morbidly sensible to light, but not injected as in phrenitis. "The pupil, as has been before observed, is always dilated in idiopathic hydrocephalus: it is only when the disease is complicated with inflammation of the brain or its membranes, that it is permanently contracted." The author attributes the occasional *spasmodic contraction of the eyelids,* resisting the exposure of the eyeball, and considered by Dr. Green as pathognomonic of the affection, to irritation of the filaments of the fifth pair supplying the orbicularis, and from the same cause there frequently exists, in connexion with it, itching of the alæ nasi and lower lobe of the ear. The auditory nerve is acutely sensible to impressions, but the olfactory becomes obtuse.

Dr. Smith believes that *cerebral auscultation* may furnish considerable assistance in distinguishing congestion, effusion, and the hydrencephaloid disease. The *inspiratory cephalic murmur* resembles a soft interrupted blowing, such as may be produced by blowing through any narrow wooden tube without compressing the lips. The *cardiac cephalic bruit* conveys "to the ear the impression of the finger gently tapping a soft elastic body, as a thin India-rubber ball, or a bladder partly filled with water. It is similar to the sound heard in the femoral artery, in a plethoric subject, on lightly applying the stethoscope." 3. The *vocal cephalic resonance* is sharp and piercing, imparting a vibratory sensation to the hand and ear. 4. The *cephalic sound of deglutition* is of a dull massive liquid character.

"When disease affects the brain or its involucra, a sensible alteration is manifest in the cephalic sound of the heart and the voice. The former, especially, notifies the commencement or existence of some change in the normal condition of the brain, when it alters its character, from a soft and feeble to a rough, harsh, or blurting sound, which in the hydrencephaloid disease is accompanied with a musical intonation of the arteries, very easily recognized and remembered when once heard. This sound is of varying intensity, according to the activity of its cause, and may be imitated by drawing the finger quickly and heavily across a piece of velvet or fustian. It is loudest in cerebral congestion, apoplexy, and in the stage of effusion, when the circulation is abnormally slow, and then not infrequently becomes rough, harsh, grating, rasp-like. In simple excitement or erethism of the brain, in commencing hydrocephalus and phrenitis, this sound is softer, quicker, more like the bellows-sound of the heart, heard in endocarditis. * * * * * * Should any thin fluid have accumulated in the head, a modification is heard in the sound of the voice transmitted through the osseous parietes of the cranium; the nature of this sound is trembling, sharp, and bleating, and it has a silvery tone not unlike the one produced in pleuritic effusions, and it may with propriety be termed the cephalic ægophony. It is very easily discerned in large aqueous collections of the skull, where the fluid is dispersed over the whole surface of the brain, and also in those cases of chronic hydrocephalus where the ventricles are considerably distended with watery accumulations. This sound may likewise be recognized in the stage of effusion in acute hydrocephalus and inflammation of the brain, &c.: but it is then less defined, becoming more sharp, clanging, vibratory. In sanguineous extravasations, the voice appears increased in intensity, but little altered in its sonorous properties. * * * * * The physical signs of the head, whilst they are not pathognomonic of any individual affection of the brain, are nevertheless exceedingly useful in assisting us to decide upon the existence, or otherwise, of disease located there or elsewhere; also whether serous effusion shall have taken place or not, and how far the disorder is one of the head only, or complicated with disease of the respiratory organs. Though I have for years attentively studied the auscultic phenomena witnessed in common in various lesions of the brain, I cannot satisfy myself, that as yet we possess any definite knowledge, which may be said to indicate with certainty, any individual or peculiar malady of that organ; but they are merely descriptive of sundry morbid changes occurring in that viscus, the result of numerous causes, whose separate effects are not at present appreciable to our senses, or distinguished by their means one from another." P. 41.

*Etiology.*—Dr. Smith rejects the doctrine of the hereditariness of hydrocephalus, and attributes its production to the various adverse hygienic circumstances influencing the child both prior and subsequent to, its birth.

" The latent causes of Hydrocephalus I take to be a strumous or lymphatic constitution, engendered not infrequently by a too highly excitable condition of the nervous system in the mother, which deprives the fœtus in utero of its due supply of nutrient aliment. This serous habit of body may also result from the want of suitable dietary, clothing, and out-door exercise, so that any previous morbid impression made upon the infantile frame acquires from these causes increased activity. That it mainly depends upon ourselves, and our modes of living to avoid the numerous ills to which flesh is heir; and that remedying our own bad habits, improving our dwellings, and paying more rigid attention to the quality of the food we eat, are capable, in the majority of instances, of eradicating and preventing a host of formidable maladies, amongst which diseases of the nervous system occupy a prominent place, is indisputably proved by the facts collected by numerous authors who have minutely investigated the subject of infantile Hygiene." P. 47.

Subscribing to the truth of the above, we still demur to the statement of its not being hereditary. Indeed, when the author allows the existence of a " family predisposition" in some instances, we think he admits enough; for no one can pretend that more than the disposition is transmissible. In fact, believing that hydrocephalus, as distinguished from phrenitis, is generally a tuberculous or strumous disease, we also believe the proof of its transmission is as strong and no stronger, as is that of the transmission of any other form of scrofula or tubercle. The author properly dwells upon the necessity of parents, especially mothers, conforming to the laws of hygiene, if they wish to rear healthy offspring; and indeed little excuse can now be found for those who persist in violating these, after the rationale of the generation of phthisical disease in the offspring of the dyspeptic, so ably delivered by Sir James Clark.

In corroboration of the propriety of placing hydrocephalus in the category of tuberculous diseases, the age at which it usually occurs may be cited. Of 155 cases, collected by Dr. Green, 45 were aged from 2 to 4 years; 54 from 5 to 7: 29 from 8 to 10: 22 from 11 to 13, and but 5 above 13. The greater number being thus aged from 5 to 7, which is the period at which tubercle according to Papavoine's researches most usually shows itself in children. MM. Rilliet and Barthez observe, that children are especially liable to hydrocephalus between 6 and 10; then from 3 to 5; then from 11 to 15; and least so from 1 to 2. Uniting their cases with those of MM. Guersant and Piet, they furnish the following numbers. Before 1 year, 3 cases; between 1 and 3, 7; between 3 and 5, 45; between 6 and 10, 84; and between 11 and 15, 29.

*Morbid Anatomy.*—To this Dr. Smith is disposed to attach no importance, believing the disease to be an idiopathic nervous fever, during the course of which inflammatory action may or may not be set up in the head—the instances being very numerous in which neither it or consequent effusion is discoverable after death.

"To enumerate a variety of cases, in which limpid effusion, softening, slight opacity of the membranes, either on the surface of the membranes, above or at the base of the brain, the bronchial, thoracic, or abdominal signs of tubercular deposit, or granular disease, is, in this view of the pathology, less important, and almost unnecessary. For I should no more expect to arrive at just notions of the pathological condition of its admonitory and subsequent stages, by meditating upon the accumulated and curious statistics furnished by necropsy, than I should expect to arrive at a true knowledge of the essence of any idiopathic fever, simply by contemplating its ravages after death." * * * * * * * * "In the gradually advancing condition of more perfect ossification, when the more intimate continuity of structure between the dura mater and the pericranium is in a great degree cut off, Hydrocephalus is said no longer to beset the path of the juvenile adult, simply because, although the adult may have the same condition of blood, the same strumous diathesis, and the same high susceptibility of nervous system, he will not have the readily yielding texture of the soft contents of the cranium, nor the same facility of hyperæmial condition of the external and internal blood-vessels of the cranium, and, therefore, not manifesting those early symptoms which Dr. Cullen denominated Hydrocephalic apoplexy. The fever of such adults will maintain the name of febris lenta nervosa, or pure nervous fever. All that has been said touching the contradictory phenomena of inflammatory appearances, confidently adduced as a set-off against those innumerable cases in which such appearances have been wanting, serves only to confirm this opinion; and is so far a proof that Hydrocephalus is not in essence an inflammation, much less a dropsy; but that it may or may not be accompanied with inflammation in its progress,—may or may not lead to effusion in its progress, just like any other idiopathic fever." P. 68.

This generalization will, we believe, be admitted by few, although most will also allow that its opposite, the doctrine of Dr. H. Green and the French School, that a deposition of tubercle in the meninges is an essential character of the disease, has been also too broadly stated.

*Treatment.*—In detailing the various rules for the preservation of public and personal health Dr. Smith reiterates his claim to an originality which cannot be allowed. "In truth, the ground is left to me without a competitor, no one having thought it worth his while to be at once an instructor and admonitor of mankind!" Other "instructors and admonitors" have wisely directed their attention, not to the preservation of health from this or that disease, but to the fortifying it from attacks, come they from what quarter they may; and so many "competitors" of name and note are there in this useful work, and so wide has information been diffused by them, that we much fear their advice and suggestions having hitherto been so imperfectly followed, has not arisen in many cases from a want of a due sense of their importance, but from the anomalous and entangled condition of our present social system presenting great obstacles to the realization of many advantages and improvements, apparently of easy attainment. Much, however, capable of accomplishment remains yet to be done; and we welcome our author as a worthy labourer in so useful and fertile a field, although we should have been better pleased had he done some homage to the numerous sowers and reapers who had pre-occupied and prepared it.

When applied to furnish *prophylactic treatment* for children suffering but trifling indisposition to appearance, but in whose family the disease has already manifested itself by attacking some of the other children, the author judiciously recommends due attention to be paid to the alvine, renal, and cutaneous secretions, to which end he prescribes measures which will suggest themselves to all practitioners. He also employs, what he terms "a revulsion bath"—a bath so contrived (an engraving of it is furnished) that cold water may be poured upon the head while the rest of the body is in contact with heated water. He also recommends some form of counter-irritation to be applied to the nape or behind the ears.

For the *treatment* of the disease when developed, Dr. Smith states the same indications present themselves as in the fevers of adults.

"If a few cases will bear blood-letting, and may be promptly arrested by it, there is nothing new in the observation or the practice, for all our predecessors have recorded this as a noticeable fact, with regard to fevers in general. From the period that this disease sets in, the chain of febrile symptoms is as little interrupted as in any idiopathic continued fever known in our climate. * * * * * * * Therefore it is that I say, treat Hydrocephalus as you would any other nervous fever, and such treatment will give you the best chance of success. If it be characterized by strong inflammatory signs about the head, by deep-coloured urine, strong pulse, and steadily increased heat of surface, injected tunica conjunctiva, urgent thirst, suffused face, and, above all, by disposition to violent restlessness, simulating in a degree that of phrenitis, blood-letting is just as admissible as it may be in a typhus, a synochus, a miliaria, or a lingering nervous fever, under the like modifications. If, happily, now and then, such treatment may, within the first forty-eight hours, arrest the mischief, so also it may in inflammatory typhus. If, however, while the general condition of the blood-vessels indicate debility, that of the cerebral vessels shows distension or oppression, in the sinus system especially, a few leeches may be admissible. So also in other fevers. Or if debility, nervous irritation, and struma, are the characteristics of our patients, neither general nor topical depletion will offer reasonable expectation of benefit. The revulsion bath alluded to, and already described, should be employed twice a-day; or the sponge-cap,—formed of pieces of thin sponge, sewed together, and having a leathern strap as a border to secure it,—may be used to cool the head, spirituous, or freezing, mixtures being employed to moisten it." P. 162.

The author recommends "a *vomit* of ipecacuanha, worked off with mustard whey," to be given two or three evenings in succession, after the first appearance of the disposition to natural vomiting. Sustained rather than violent *purging* should be instituted; commencing with calomel and jalap, and then resorting to cream of tartar, toasted jalap, or castor oil, "nor should a day pass without administering a clyster, in which, and with the purgatives, as in nervous fever, or subsultus, I would combine valerian, castor, assafœtida, and other remedies of that class." Just as in other fevers, also salines and diuretics may be given, as also small doses of mercury, not, however, with any intention of producing its specific effects. The diet must be regulated also as in fever; and, prior to advanced effusion, small doses of laudanum, syrup of poppies, Hoffmann's anodyne, &c. may be given to calm irritation. In the second and third stages counter-irritation is indicated.

"From the foregoing observations, it will appear that the principal indications to be fulfilled in the treatment of this malady are, if possible, to produce a favourable crisis, by the steady and free action of the abdominal or cutaneous secretions. Hence free catharsis, free diuresis, or free diaphoresis, by whatever means these separate states of the system can be obtained, offer the most reasonable prospect of recovery. The employment of specific medicines, such as bicloride of mercury, iodine, tincture of Spanish fly, tartar emetic, &c. appears to me to be founded on erroneous impressions, touching the nature and essence of the disorder, and in the majority of cases will be utterly inadequate to the end proposed." P. 166.

The analogy here attempted to be traced between the hydrocephalus of children and the fevers of adults is defective, and in no particular more so than in the important one of the result of treatment, as exemplified in the infinitely greater mortality of the former disease, compared with that of the latter. In fact, hydrocephalus is more easily confounded with other diseases than the author's directions to parents and nurses would lead one to expect; and, however some of these febrile and inflammatory affections of infancy might be benefited by the treatment above suggested, we should entertain little hopes of the genuine disease being thus vanquished. It is easier to criticize one mode of treatment of the disease than to propose another more likely to be of avail. This we frankly confess we are unable to do, and much fear that it is but too true, that hydrocephalus once developed is seldom if ever curable. In proportion as correct ideas of the pathology of the disease—its intimate connection with, if not its absolute dependence upon, the strumous constitution—have prevailed, has confidence diminished in that mode of procedure which would treat it as a purely inflammatory affection. We approve of the moderation and discrimination as regards blood-letting, inculcated by Dr. Smith; for, although we are far from participating in the unreasonable fears of bleeding children expressed in Mr. Hood's recent work, and believe that the saving their lives often depends upon this being abundantly and promptly resorted to, yet we feel certain that hydrocephalus is not one of the diseases in which recourse can be had freely to it. The excessive depletion advised by Maxwell, Davis, and others, is quite inappropriate in genuine examples of this affection. Phrenitis and arachnitis have been, in some instances, doubtless mistaken for it by these authors; and in too many others, cephalgia and febrile action, proceeding from more innocent causes, have been hastily assumed as indicative of hydrocephalus; and although they would have yielded to moderate measures, have been unnecessarily and injuriously submitted to this "heroic" treatment.

